# Social Progress in Spanish Municipalities (2001–2011)

**DOI:** 10.1007/s11482-016-9502-7

**Published:** 2016-12-27

**Authors:** Ana Cárcaba, Eduardo González, Juan Ventura

**Affiliations:** University of Oviedo, Av. Cristo s/n, 33006 Oviedo, Spain

**Keywords:** Quality of life, Spain, Municipalities, DEA, Malmquist

## Abstract

This paper proposes a methodology for the assessment of social progress in the biggest Spanish municipalities between years 2001 and 2011. We follow recent descriptions of QoL to elaborate a measurement framework composed of eight dimensions, for which 16 subindicators are elaborated from information collected using different data sources. Weight constrained Data Envelopment Analysis is used to estimate QoL composite indicators in both periods and to compute a Malmquist index of social progress, which assesses the evolution of the indicators during the decade. The results evidence positive social progress with an average improvement of about 5% during the decade. While the Central-Northern regions still show the highest levels of QoL, the Southern regions (including the islands) dominate the improvement trend. We then decompose the Malmquist index into a catching-up effect and a frontier shift effect. Positive catching-up is measured in almost all the regions. The worst performing municipalities in 2001 experienced the largest catching-up effects, a trend that contributes to territorial convergence. The frontier shift also shows a positive trend.

## Introduction

While social progress has been traditionally associated with economic macro-indicators, social science today is concerned with the need to complement these indicators with other critical domains of well-being, such as health, education or environmental factors (Costanza et al. 2009; Fitoussi and Stiglitz 2011). Introduced in 1990, the Human Development Index was the first notable attempt to construct a composite indicator of Gross Domestic Product (GDP), Health and Education, in order to assess social progress in developing and underdeveloped countries. The interest on the topic increased rapidly during the 1990s and 2000s and peaked after the influential report of the Commission on the Measurement of Economic Performance and Social Progress (Stiglitz et al. 2010).

Regarding the unit of analysis, applied research in the measurement of QoL has focused mainly in countries and individuals.^1^ The interest in comparing the QoL in different countries is evident and today we count with statistical information available at this level of analysis. In contrast, within country analyses of QoL are far more difficult to carry, since statistical information on the many domains that contribute to QoL is not readily available at the local level. The indicators required are only available for the biggest cities or for aggregated territories such as regions or provinces. This is unfortunate, since it has been shown that the local level (municipality) may be more relevant in the assessment of the QoL than the regional or provincial levels (González et al. 2011).

In the case of Spain, an important part of the statistical information required for constructing a composite indicator of QoL at the municipal level can be obtained from the census, which is elaborated from decade to decade. González et al. (2011; 2016) used this information, together with some other statistics, to estimate QoL scores for a comprehensive sample of Spanish municipalities in 2001 and 2011, respectively. In the present paper we use these data in order to track the evolution of QoL in Spain during the decade 2001–2011 by computing composite indexes of social progress. The economic impact of the financial crisis (started in 2008) in Spain has been extensively analysed recently (Guardiola et al. 2015; Méndez et al. 2015). With negative growth of GDP (from 2009 to 2013) and alarming unemployment figures (peaking 27% as of January 2013), the risk of poverty and social exclusion increased dramatically in Spain after the crisis. It is estimated that 20% of the Spanish population was below the poverty line in 2013, five points more than in 2004.^2^ The severe material deprivation rate also rose from 4.8 in 2004 to 6.2 in 2013.^3^ However, the QoL construct expands in other dimensions which are not completely correlated with economic macro-magnitudes. In order to track the evolution of QoL in the Spanish municipalities we have carefully collected a comprehensive set of social and economic indicators covering all the relevant dimensions of QoL in 2001 and 2011.^4^

We rely on Data Envelopment Analysis (DEA) for the computation of the composite indicator of QoL. The DEA methodology generates internal weights for the aggregation of the QoL domains considered into a single composite index. While DEA was not initially designed for the measurement of the QoL, after the pioneering work of Hashimoto and Ishikawa (1993), its use within the social indicators literature has become increasingly popular (Mariano et al. 2015). The DEA methodology can also be extended to track the temporal evolution of QoL. The Malmquist index, originally developed for the assessment of the temporal evolution of firm productivity (Caves et al. 1982), can also be employed to track the change in QoL between different periods (Hashimoto et al. 2009; Carboni and Russu 2015). Furthermore, the Malmquist index can be decomposed into two different sources of time variation: a frontier shift and a catching-up effect. The frontier shift will show the part of the variation in QoL that can be considered common across municipalities. In contrast, the catching-up effect will capture the particular evolution of each municipality with respect to the joint evolution of the other municipalities. Ideally, both effects should be positive, representing collective social progress and a trend towards convergence, respectively. However, negative shocks such as an economic downturn may provoke social regress instead of social progress. The computation and decomposition of the Malmquist index will shed light into this issue for the recent evolution of the Spanish municipalities.

The paper is structured as follows. “The dimensions and measures of Quality of Life” section briefly reviews the literature on the measurement of the quality of life. “Methods” section describes the data sources that can be employed to measure QoL in Spanish municipalities and contains our proposed framework. “Results” section describes the weighted constrained DEA model used and the computation and decomposition of the Malmquist index of social progress. Finally, “Concluding remarks” section discusses the main results obtained.

## The Dimensions and Measures of Quality of Life

The flaws of GDP are well known to economists and social scientists (see Stiglitz et al. 2010). Therefore, it should not be used as an unquestionable and unique guide for policy making, as is often done in economic and political analysis. Human development and well-being emerge as broader goals for society which correlate only imperfectly with GDP, as noted early by Easterlin (1974) and others (Campbell et al. 1976; Andrews and Withey 1976). Furthermore, while it can be shown that human development has a positive impact on economic growth, the opposite is not necessarily true (Ranis and Stewart 2000). Quality of life composite indicators aim at summarizing varied information about the many different dimensions of life that drive welfare.

With the turn of the century, several operative proposals have gained influence in the field of QoL measurement. The influential report of the French Commission on the Measurement of Economic Performance and Social Progress (CMEPSP), elaborated by Stiglitz, Sen and Fitoussi in 2009, highlighted the multidimensional nature of QoL and sustainability and specified the type of statistical information that should be developed in order to obtain useful indicators. Several institutions took the challenge of developing such indicators, most notably the OECD and the European Statistical System (ESS). Since 2013, the OECD publishes the Better Life Index and How is Life, addressing quality of life along 11 dimensions (housing, income, jobs, community, education, environment, civic engagement, health, life satisfaction, safety and work-life balance). In turn, closely following the CMEPSP recommendations, the ESS Sponsorship group on Measuring Progress, Well-being and Sustainable Development, recommended 8 + 1 dimensions along which QoL should be addressed (material living conditions, productive or main activity, health, education, leisure and social interaction, economic and physical safety, governance and basic rights, natural and living environment, overall experience of life).

These efforts are notable and constitute a critical improvement in the assessment of QoL in Europe. Unfortunately, the development of statistical information is still far from reaching the municipal level of analysis. Local information about the different dimensions of QoL is scant and dispersed within Europe. A remarkable contribution at the local level is the Urban Audit Project (UAP), started back in 1999. The UAP compiles data in 9 dimensions (demography, social aspects, economic aspects, civic involvement, training and education, environment, transport and travel, culture and leisure, innovation and technology) with more than 300 variables corresponding to 284 European cities. The scope of the project is however limited, since only the biggest European cities are included in the database.

Despite these data limitations, the assessment of QoL in cities is gaining academic interest (Ballas 2013). Some international early examples include estimations of QoL for US metropolitan areas (Becker et al. 1989), Japanese prefectures (Hashimoto and Ishikawa 1993) or US counties (Marshall and Shortle 2005). Within Europe, Morais and Camanho (2011) used the Urban Audit data to compute composite QoL indicators for an extensive sample of 206 cities belonging to 25 countries. In contrast, within country studies in Europe are still scant. Bigerna and Polinori (2013) in Italy, Poldaru and Roots (2014) in Estonia and Murgaš and Klobučnik (2016) in the Czech Republic are recent examples. In the case of Spain, the most comprehensive study measured QoL in a sample of 643 municipalities for 2001, covering 75% of the Spanish population (González et al. 2011). More recently, González et al. (2016) have estimated QoL indexes for a sample of 393 municipalities in Spain in 2011. Other authors have estimated QoL indexes for smaller intraregional samples, including Martin and Mendoza (2013) for Canarias, Royuela et al. (2003) for the province of Barcelona, Zarzosa (2005) for the province of Valladolid or López and Sánchez (2009) for Galicia.^5^ QoL has also been estimated indirectly from the analysis of inter-municipal migration patterns (Navarro and Artal 2015).

While there is no complete consensus about the exact specification of the dimensions that must be taken into account in measuring QoL, the guidelines provided in the Stiglitz et al. (2010) report and the subsequent work of the ESS Soponsorship group and the OECD’s “Better life” initiative have a high degree of commonality. Following these proposals, González et al. (2016) elaborated an integrative framework that considers 8 dimensions^6^ (Table 1).

**Table 1:** Eight dimensions of QoL

	Our proposal	Stiglitz et al. (2010)	Sponsorship group	OECD
1	Material living conditions	Economic insecurity	Material living conditions	Income, Housing
2	Health	Health	Health	Health
3	Education	Education	Education	Education
4	Environment	Environmental conditions	Natural & living environment	Environment
5	Economic & physical safety	Personal insecurity	Economic & physical safety	Safety, Jobs
6	Governance & political voice	Political voice & governance	Governance & basic rights	Civic engagement
7	Social interaction	Social connections	Leisure & Social interaction	Community
8	Personal activities	Personal activities	Productive & valued activities	Work-Life balance

As noted previously, data availability is a major limitation for the assessment of QoL at the local level of analysis. In particular, it requires combining many different sources and elaborating partial indicators from microdata that were originally compiled for other purposes. One of the principal sources of information at the municipal level is the census, which is elaborated every 10 years by the Spanish *Instituto Nacional de Estadística* and contains varied information about the people and the dwellings. Only the municipalities over 20,000 citizens are identified in the 2011 census microdata, which limits our study to a sample of 393 municipalities. Other alternative sources were also used to obtain information about mortality rates, crime, pollution records, volunteering activities and governance. From these sources, a battery of indicators were developed in order to account for each of the eight dimensions of QoL at the municipal level.^7^

### Material Living Conditions

While we don’t have information on per capita income at the municipal level for the entire sample, the census microdata provides a good proxy that is called Average Socioeconomic Condition (ASC). This variable is based on the occupation of every individual over 16. A scale from 0 to 3 is used to associate occupations with the corresponding socioeconomic status of the individual. Its municipal average is a good indicator of material living conditions. A second element related to this dimension is housing, which is also partially associated with health concerns. From the census microdata we computed the Average Net Surface (ANS) and the average Living Conditions of the Dwellings (LCD).^8^ By multiplying both variables we computed a combined indicator of the overall Quality of the Dwellings (QD = ANS · LCD).

### Health

We worked with mortality microdata to construct two indicators that reflect health differences within the Spanish territory.^9^ The first indicator we constructed is Excess of Mortality (EM) adjusted by age. To construct this indicator for each municipality, we divided the population into age groups of 5 years (0–5, 6-10….) and then computed mortality rates within each age group.^10^ These rates were adjusted by weighting each age group rate by the national norm. The age-adjusted mortality rate of the municipality was then divided by the aggregate national mortality rate. This ratio reflects whether age-adjusted mortality in the municipality is higher or lower than the national norm. Then, we constructed a second indicator using mortality microdata called Avoidable Mortality (AM). We counted the number of deaths that can be classified as avoidable following a consensus of Spanish health experts (Gispert et al. 2006). Our AM variable is the ratio of avoidable deaths to total population in the municipality.^11^ These variables should reflect the health outcomes derived from life habits (such as alcoholism, sedentary lifestyles, or smoking) and the quality in the functioning of the health system in the territory (including active preventive health activities).

### Education

Education increases subjective QoL (Ross and Van Willigen 1997) and additionally generates positive externalities on the community (Grace 1989). Therefore, it is not only the own education level what influences QoL but the joint education level of the community. The census microdata contain two relevant indicators of educational attainment. The first, and most informative one, is the overall level of education (OLE), in a scale from 0 (illiterate) to 10 (PhD). The census also provides a dummy variable indicating whether the individual completed a university degree (UD) or not. These data are registered for population over 16 and our variables are the population averages.

### Environment

The Spanish Ministry of Agriculture, Food and Environment publishes data on the quality of air, obtained from a network of stations for air quality measures. We compiled data on two different pollutants which are subject of big concern for health according to the World Health Organization (WHO 2006): 1) Particulate matter (PM_10_, average daily value), which, according to the WHO, affects more people than any other pollutant, and 2) Ozone (O_3_, 26^th^ maximum 8-h mean).^12^

### Economic and Physical Safety

A usual indicator of economic safety is the Unemployment Rate (UR), a well-recognized source of economic insecurity and social exclusion. Further, unemployment is associated with a deterioration of physical and mental health (Lahelma 1992; Janlert 1997) and psychological well-being (McKee-Ryan et al. 2005). People who become unemployed report lower subjective quality of life even after controlling for the loss of income (Fitoussi and Stiglitz 2011). Physical safety is also important, not only because of its most obvious effect on physical integrity, but also because of the effect of perceived insecurity in emotions (Stiglitz et al. 2010). Upon request, the Spanish Ministry of Home Affairs provided disaggregated crime data for all the municipalities in the sample except those in País Vasco and Cataluña. Unfortunately, for these two regions we only had access to aggregate data.^13^ Therefore, we use the total number of crimes divided by total population (CRI).

### Governance and Political Voice

The quality of local governance greatly affects the quality of the public services received by the citizens and, therefore, is of paramount importance to QoL. The financial condition of the local government can be used as a proxy of the quality of public management (e.g., Groves et al. 1981; Zafra-Gómez et al. 2009; Cuadrado-Ballesteros et al. 2012). Along this line, the financial result or cash surplus is a key indicator of financial health. In order to avoid the size effect in this indicator, we took the ratio of the cash surplus on the total budget of the local government (CS). In the same way, active participation of citizens in public decision making is a sign of freedom and concern about the community. Political voice is also critical for public policy accountability. The only available indicator of political voice for the whole sample of municipalities was the percentage of participation in municipal elections (PME). Voter turnout is a common indicator for this dimension and has been used, for instance, in the OECD’s better life index within the civic engagement and governance dimension.

### Social Interaction

The existence of places and institutions that facilitate social interaction can be beneficial to QoL since they ease developing social and cultural relations (Lloyd and Auld 2002). Involvement towards the community is also an important part of social interaction that contributes to QoL. Two indicators are available to be used as proxies for this dimension. The first one, included in the census microdata, is the participation in volunteering activities (VA), which shows the degree of commitment with the most needed in the community. The second variable is the total number of cultural and social centers available in the municipality, divided by the population (CSC).^14^

### Personal Activities

Related with the previous dimension is the time devoted to non-working pleasant activities. This is a very difficult dimension to measure with objective data, since it would also require subjective information about the satisfaction with the activities. Our municipal database contains two variables that reasonably approximate this dimension. The first one is the commercial market share (CMS), a variable included in the Anuario Económico de España 2011 which is elaborated by La Caixa.^15^ This variable indicates the proportion of commercial activity that takes place within the municipality boundaries in relation to the total commercial activity of Spain. The second proxy is commuting time (CT), which negatively affects QoL since it withdraws time from pleasant personal activities.^16^ Commuting has been consistently associated with reduced subjective well-being even after compensating for the corresponding increase in income (Stutzer and Frey 2008).

Table 2 shows the complete list of indicators used to approximate the 8 dimensions of QoL.^17^ Unfortunately, for two of these indicators (CS and VA) we were unable to collect comparable data for 2001, since this information was not publicly available in that year. We performed two different analyses, one considering that these two indicators have not changed during the period (i.e., using the same data of 2011 for 2001) and another one excluding these two indicators from the analysis.^18^

**Table 2:** Partial indicators of the QoL dimensions

QoL dimension	Indicators
Material Living Conditions	Average Socio-economic Condition (ASC) Quality of Dwellings (QD)
Health	Excess Mortality (EM)I Avoidable Mortality (AM)
Education	Overall Level of Education (OLE) Population with a University Degree (UD)
Environment	Particulate Matter (PM_10_) Ozone (O_3_)
Economic & Physical Safety	Unemployment Rate (UR) Crime rate (CRI)
Governance & Political voice	Local government Cash Surplus (CS) Participation in Municipal Elections (PME)
Social interaction	Population participating in Volunteering Activities (VA) Cultural and Social Centers (CSC)
Personal Activities	Commercial Market Share (CMS) Commuting Time (CT)

## Methods

In order to compute the Malmquist indexes of social progress, the first step is to estimate the composite indicators of QoL for 2001 and 2011 on the basis of the 16 partial indicators listed in Table 2. Of the different methodologies suggested by the OECD’s Handbook on Constructing Composite Indicators (Nardo et al. 2005), we have followed the non-parametric Data Envelopment Analysis (DEA). This technique introduces a very conservative approach to weighting, in which the algorithm empirically determines the weights of the different partial indicators of the composite indicator. It is known as a Benefit of the Doubt (BoD) approach, since the weights determined are those which are most favourable to the municipality under analysis (i.e., those that maximize the value of the composite indicator). The application of DEA to the measurement of QoL was first proposed by Hashimoto and Ishikawa (1993) and has been extensively used since (Mariano et al. 2015). However, this BoD approach has been seriously challenged for producing inconsistent or “false” results, especially for the high end of the QoL spectrum (Sharpe and Andrews 2012). The use of weight restrictions may introduce the necessary consistency in the computations, while assuring some desirable degree of flexibility at the same time.

Let’s start with the DEA traditional specification of Charnes et al. (1978) with a ratio form:

1 $$ \begin{array}{l} \min \kern0.5em \frac{{\displaystyle \sum_{m=1}^M{v}_m{x}_{im}}}{{\displaystyle \sum_{s=1}^S{u}_s{y}_{is}}}\\ {}s.a:\\ {}\frac{{\displaystyle \sum_{m=1}^M{v}_m{x}_{jm}}}{{\displaystyle \sum_{s=1}^S{u}_s{y}_{js}}}\ge 1\kern1em ,\kern1em \forall j\\ {}{u}_s,{v}_m\ge 0\kern1em ,\kern1em \forall s,m\end{array} $$

where *x*_*im*_ represents the amount of input *m* in municipality *i*, *y*_*is*_ represents the amount of output *s* in municipality *i*, *v*_*m*_ is the weight of input *m, u*_*s*_ is the weight of output *s* and *j* represents any of the municipalities in the sample.^19^

While using the standard DEA model to estimate QoL (using city amenities as outputs and city drawbacks as inputs) is very straightforward, that approach is indeed problematic. The DEA model may not be neutral to the selection of dimensions as inputs or outputs, which in this setting is certainly arbitrary. For instance, we can use the same raw data to compute either crime rates (input) or safety rates (output). To avoid this problem, we transformed all the initial variables into outputs (i.e., more is better). In doing so, we followed the distance to the group leader normalization method proposed by Cherchye et al. (2004). In the case of goods, this method implies dividing the value of the variable by its maximum (ASC, QD, OLE, UD, CS, PME, VA, CSC and CMS) and, in the case of bads, the minimum of the variable is divided by its value (EM, AM, PM_10_, O_3_, UR, CRI and CT). The transformed variables vary from 0 to 1 and higher values indicate better QoL. While applying this transformation, we noticed it is very sensitive to abnormal values in the minima or maxima of the variables. In order to reduce the effect of single observations on all the values of the transformed variables, we decided to substitute the maxima and minima by the 1% and 99% percentiles, respectively. In the case of municipalities below the 1% percentile or above the 99% percentile, we substituted the value by the corresponding percentile value, in order to assure the range of variation to be within the (0,1] interval.^20^

The resulting model is equivalent to the estimation of the following composite indicator (Cherchye et al. 2007):

$$ \begin{array}{l} \max \kern0.5em {\displaystyle \sum_{s=1}^S{u}_s{y}_{is}}\\ {}s.a:\\ {}{\displaystyle \sum_{s=1}^S{u}_s{y}_{js}}\le 1\kern1em ,\kern1em \forall j\\ {}{u}_s\ge 0\kern1em ,\kern1em \forall s\end{array} $$

This program finds the weights *u*_*s*_ that maximize the QoL composite indicator for municipality *i*. If municipality *i* is on the QoL frontier, then the objective function will reach the maximum possible value of 1. Conversely, non-frontier municipalities will obtain maximum values lower than 1. This means that, even with the most favourable set of weights, there is at least other municipality that obtains a higher weighted sum. The composite indicator is bounded within the (0,1] interval, with values lower than 1 reflecting the distance to the QoL frontier. The former mathematical program is equivalent to a standard DEA specification which includes a fictitious input dummy variable taking the value 1 for all municipalities.^21^

Given that the linear program is computed independently for each municipality *i*, it may (and will) happen that the set of optimal weights be completely different across them. Furthermore, many of the optimal weights will take the value 0, which is a signal that the municipality is not comparatively good in that dimension. In other words, the value of the municipality in that dimension would be totally irrelevant in the final value of the composite indicator, which is not reasonable and constitutes a well-known inconsistency of the DEA method. Many different solutions have been suggested in the literature, which imply restricting the range of acceptable values for the weights (Thompson et al. 1986; Dyson and Thanassoulis 1988; Allen et al. 1997; Roll et al. 1991; Wong and Beasley 1990; Pedraja et al. 1997; Halme et al. 1999; Sarrico and Dyson 2004).

A controversial issue in the weight restriction literature is how to determine the acceptable range of weights. The goal of using weight restrictions should be to combine the virtues of flexibility (being unrestricted DEA the most flexible alternative) with an appropriate degree of consistency that guarantees that all the dimensions are reasonably taken into account (being equal weighting the alternative that better guarantees this). In this paper, we propose following a classic weight restrictions scheme, which combines a degree of flexibility with an equivalent degree weight consistency. The basic idea is to impose 50% consistency, while still allowing for 50% flexibility. This balanced trade off can be achieved by imposing the constraint that each partial indicator has at least one half of the weight it would have under an equal weighting scheme and no more than one half more.^22^ Given that we use 16 partial indicators, we added the following constraint to the mathematical program for each partial indicator *k*:

$$ 0.03125\le \frac{u_k}{{\displaystyle \sum_{s=1}^{16}{u}_s}}\le 0.09375\kern1em ,\kern1em k=1\dots 16 $$

This linear program can be run alternatively with the data of 2001 and with the data of 2011, obtaining the QoL scores for 2001 and 2011, respectively. However, the comparison between these two indicators cannot be interpreted as a measure of social progress (or regress) during the decade. The direct comparison of the QoL scores of 2001 and 2011 would only indicate how the municipality has come closer to or dropped away from the frontier, but not how much progress (regress) is observed. The reason is that the QoL frontier is also moving as a consequence of social progress (or regress). Within the DEA methodology, the correct way to estimate the overall improvement of each municipality is by computing the Malmquist productivity indexes introduced by Caves et al. (1982). In its original context of firm productivity, the Malmquist index would measure the overall improvement in the productivity of a production process between period *t* and period *t + 1*. In our context it will denote the overall social progress (regress) between both periods.

Let’s denote the DEA distance function estimated with the data of period *t* for municipality *0* as *D*_*0*_^*t*^ *(y*_*0*_^*t*^ *)*. We may also estimate the distance to the period *t* frontier but using the data from period *t + 1* for municipality *0*, which will be denoted as *D*_*0*_^*t*^ *(y*_*0*_^*t+1*^ *)*. The Malmquist index of social progress for municipality *0* would reflect the difference between both distances, using the period *t* frontier as the reference for comparison:

$$ {M}_0^t\left({y}_0^t,{y}_0^{t+1}\right)=\frac{D_0^t\left({y}_0^{t+1}\right)}{D_0^t\left({y}_0^t\right)} $$

Alternatively, we could set period *t + 1* as the standard QoL frontier to do the comparison:

$$ {M}_0^{t+1}\left({y}_0^t,{y}_0^{t+1}\right)=\frac{D_0^{t+1}\left({y}_0^{t+1}\right)}{D_0^{t+1}\left({y}_0^t\right)} $$

Since the value of the two indexes can be different, the most common adjacent Malmquist index is defined as the geometric mean of both (Caves et al. 1982). The use of this index has been shown to be problematic, since it is not circular and is subject to infeasibilities when variable returns to scale are employed. In order to solve these problems, a different approach, called the Global Malmquist Index, was introduced by Pastor and Lovell (2005). However, this index was still subject to the problem that the values computed changed when new periods were added to the database. The Biennial Malmquis Index, developed by Pastor et al. (2011) as a refinement of the Global Malmquist Index, solved this problem. It avoids considering period *t* or period *t + 1* as the reference for comparison. Instead, the reference (biennial) DEA frontier will be constructed with the data of both periods *B* = *t,t + 1*:

$$ {M}_0^B\left({y}_0^t,{y}_0^{t+1}\right)=\frac{D_0^B\left({y}_0^{t+1}\right)}{D_0^B\left({y}_0^t\right)} $$

Following Pastor et al. (2011), the biennial Malmquist index can be decomposed into the usual efficiency change (catching-up) and technical change (frontier shift) components:

$$ \begin{array}{l}{M}_0^B\left({y}_0^t,{y}_0^{t+1}\right)=\frac{D_0^B\left({y}_0^{t+1}\right)}{D_0^B\left({y}_0^t\right)}\cdotp \frac{D_0^{t+1}\left({y}_0^{t+1}\right)}{D_0^t\left({y}_0^t\right)}\cdotp \frac{D_0^t\left({y}_0^t\right)}{D_0^{t+1}\left({y}_0^{t+1}\right)}=\\ {}=\frac{D_0^{t+1}\left({y}_0^{t+1}\right)}{D_0^t\left({y}_0^t\right)}\cdotp \frac{D_0^B\left({y}_0^{t+1}\right)/{D}_0^{t+1}\left({y}_0^{t+1}\right)}{D_0^B\left({y}_0^t\right)/{D}_0^t\left({y}_0^t\right)}=C{U}_0\cdotp F{S}_0\end{array} $$

The catching-up effect (CU) measures whether the municipality is closer to or farther from the QoL frontier in period *t + 1* than it was in period *t*. Values larger than 1 indicate that the municipality is catching-up to the QoL frontier. If the frontier remains unchanged, any improvement in the municipality will generate positive catching-up. If the frontier expands, then positive catching up will only occur if the improvement in the municipality is larger than the improvement in the frontier. Finally, if the frontier is moving backwards, then we can observe positive catching up even if there is no change at all in the municipality. As such, positive catching up will generate a phenomenon similar to the classic regression to the mean processes, reducing the dispersion in QoL within the sample.

The second component, frontier shift (FS), measures the effects of social progress or regress that move the QoL frontier outwards or inwards and therefore affect all municipalities. The ratio compares the “QoL gap” between the biennial and *t + 1* frontier with the “QoL gap” between the biennial and *t* frontier. If the ratio is larger than 1 it means that the *t + 1* frontier is closer to the biennial frontier than the *t* frontier, which reflects joint social progress. In contrast, if the FS is lower than 1, it would mean that the period *t* frontier was closer to the biennial frontier, indicating joint social regress. This would indicate that (at least in that part of the frontier) the QoL partial indicators have lower values in period *t + 1* than in period *t*. This may happen, for instance, as a consequence of an economic crisis that causes a sudden rise in unemployment and reduces income. If the other indicators (health, pollution, etc.) don’t produce a compensation enough, then FS could be lower than 1. Frontier shift will show a change in the benchmark municipalities.

We believe that the computation and decomposition of the Malmquist index can bring new important insights to the dynamic analysis of the QoL. It will show which territories are catching-up with social progress and which territories are unable to follow the expansion of the QoL frontier.

## Results

Table 3 contains descriptive statistics for 2001 and 2011 of the 16 partial indicators of QoL developed for this research. The averages and standard deviations have been weighted by the population of the municipality. The 6 first indicators listed correspond to the most classic dimensions of the QoL construct (material living conditions, health and education). All of them show a positive evolution between 2001 and 2011. Especially significant are the improvement in the percentage of the population with a university degree (UD) with a 36% increase and the reduction in the rates of avoidable mortality (AM), which dropped by 24% during the decade. Pollution records also show a positive evolution, with PM_10_ decreasing 29% and O_3_ levels showing a slight increase. The rest of the variables show moderate variation. It is notable the reduction of 15% in commuting times, which reflects improvement in transportation. An interesting reduction in inequality is also observed, since the coefficient of variation has dropped in eight variables, remained stable in two and increased in only four variables (the other two have no variation since they were not observed for 2001 as explained before). Differences in education attainment increased greatly, as reflected by the difference between the minimum and the maximum. In contrast, differences in pollution records, unemployment, crime rates, consumption or commuting reduced significantly.

**Table 3:** Partial indicators of QoL (Descriptive Statistics)

	Average		Min		Max		Coeff. Var.	
	2001	2011	2001	2011	2001	2011	2001	2011
ASC	1.00	1.04	0.63	0.72	1.27	1.24	0.10	0.09
QD	5745	6500	3520	3730	11,815	11,945	0.14	0.14
EM	1.01	1.00	0.63	0.57	1.85	1.43	0.12	0.12
AM	160.1	121.3	42.5	30.4	359.1	226.5	0.21	0.23
OLE	2.89	3.12	2.19	1.90	3.48	4.80	0.07	0.15
UD	0.16	0.21	0.04	0.06	0.46	0.51	0.41	0.35
PM_10_	38.5	27.2	12.0	8.4	89.0	46.0	0.29	0.23
O_3_	105.1	107.9	41.0	73.0	136.0	141.0	0.17	0.10
UR	13.9	14.8	5.2	6.0	41.9	29.3	0.33	0.26
CRI	59.6	58.0	5.2	12.3	368.0	244.1	0.55	0.43
CS	-	0.02	-	−1.71	-	1.34	-	12.7
PME	0.64	0.62	0.46	0.45	0.84	0.79	0.09	0.11
VA	-	0.04	-	0.02	-	0.06	-	0.18
CSC	31.9	27.9	2.0	2.2	131.8	105.6	0.34	0.36
CMS	121.2	105.9	87.8	82.3	244.2	144.1	0.09	0.06
CT	23.6	20.1	10.1	11.2	39.6	31.8	0.25	0.21

The QoL indexes and the Malmquist decomposition are presented in Table 4, aggregated for each Autonomous Community (AC).^23^ Some of these ACs are large (in terms of population) as Madrid, Cataluña, Andalucía or Comunidad Valenciana and some are small as La Rioja or Navarra. These differences are reflected in the number of municipalities from each of the ACs included in the sample, which is shown in the first column. The second column indicates the percentage of the population of the AC which lives in the municipalities included in the sample. Some ACs, such as Madrid or Murcia, are very well represented in the sample, since the vast majority of the population lives in large municipalities. The opposite occurs in more rural ACs which are not so well represented in the sample (Castilla la Mancha, Navarra or Extremadura). Globally, 68% of the Spanish population is represented by the 393 municipalities in our sample.

**Table 4:** Malmquist index of social progress decomposed

	n	Coverage %	QoL index 2001	QoL index 2011	Malmquist index	Catching-up effect	Frontier Shift
Andalucía	81	67.7	0.73	0.75	1.075	1.040	1.034
Aragón	4	58.5	0.82	0.85	1.074	1.047	1.026
Asturias	7	69.4	0.79	0.82	1.070	1.046	1.023
Baleares	12	70.8	0.78	0.80	1.065	1.037	1.027
Canarias	25	76.8	0.76	0.78	1.057	1.025	1.032
Cantabria	5	54.0	0.81	0.84	1.068	1.046	1.021
Castilla y León	15	50.8	0.86	0.86	1.021	1.003	1.018
Castilla-La Mancha	15	40.5	0.80	0.83	1.061	1.035	1.026
Cataluña	63	70.3	0.79	0.78	1.024	0.994	1.030
Com. Valenciana	63	72.0	0.78	0.79	1.049	1.022	1.026
Extremadura	7	40.1	0.84	0.86	1.054	1.027	1.026
Galicia	22	51.4	0.84	0.85	1.044	1.017	1.026
Madrid	32	90.3	0.78	0.81	1.075	1.045	1.027
Murcia	17	82.5	0.79	0.80	1.048	1.012	1.035
Navarra	3	39.4	0.90	0.91	1.035	1.013	1.021
País Vasco	18	64.4	0.86	0.86	1.031	1.007	1.023
La Rioja	2	55.2	0.92	0.91	1.014	0.991	1.023
Ceuta/Melilla	2	100	0.74	0.73	1.034	1.000	1.034
Total	393	68.0	0.79	0.80	1.054	1.025	1.028

La Rioja and Navarra are consistently the ACs with a highest average QoL in both periods, while Andalucía and Ceuta/Melilla occupy the last positions also in both periods. The Malmquist index points to important social progress during the decade. The average for Spain is 1.054, which means that aggregate QoL had increased an average of 5.4% in 2011 with respect to 2001. While all the ACs show a positive trend, according to the Malmquist index, the improvement has not been equally distributed across the territory. Andalucía, Madrid, Aragón and Asturias obtain averages above 1.07. This is interesting especially for Andalucía, which was far below average in 2001. In contrast, La Rioja, Castilla y León and Cataluña show modest progress, with averages below 1.03. This is worrying for Cataluña, since this AC had only an average performance in 2001.

We recall now that social progress, as quantified by the Malmquist index, has two markedly different components. The first one is the general trend of the QoL frontier, which may be either expanding or contracting. The second one is the particular movement of each municipality towards the frontier. Of course, in order to be closer to the new QoL frontier, progress in a municipality must be greater than the shift of the frontier. The last column in Table 4 shows the average frontier shift for municipalities. On average the frontier has expanded by 1.028 (i.e., by 2.8%). Even though this shift is not identical in all parts of the QoL frontier the averages for the different ACs are very similar, ranging from 1.035 in Murcia to 1.018 in Castilla y León. For most ACs, the frontier shift is around 1.025. Those municipalities that are able to improve living conditions by this rate will maintain their relative position with respect to the QoL frontier. Therefore, social progress may come without an increase in the QoL index (which is relative to the frontier). The QoL index will only increase if social progress in the municipality is greater than social progress in the frontier. If that is the case for the municipalities with a lower QoL score in 2001, then positive catching-up occurs and the distribution of QoL would accumulate mass next to the value 1.

Table 4 shows that this is indeed the case in our sample. The national average catching-up is 1.025, which means that, in 2011, the average municipality was 2.5% closer to the frontier than it was in 2001. This is an impressive result, since, as we have just discussed, the frontier has moved outwards by an average of 2.8%. Again the distribution of the catching-up effect is not even across the territory. Aragón, Asturias, Cantabria, Madrid and Andalucía show averages above 1.04, while La Rioja and Cataluña show slightly negative catching-up with averages of 0.99. In general, we observe that the municipalities that scored lower in 2001 have the highest catching-up, which is a very positive result from the viewpoint of social cohesion. The correlation between the index of QoL in 2001 and the catching-up effect is −0.49 and is statistically significant at the 0.01 level, which confirms this observation. The correlation coefficient between the QoL score in 2001 and the Malmquist index is even larger in absolute terms (−0.56), therefore confirming a positive trend in the reduction of territorial inequality in the distribution of social progress.

The distribution of weights for the 16 variables in both periods is shown in Table 5. Some indicators (ASC, O3, CMS, CT, PME and CS) receive large average weight shares in both periods. In contrast, other indicators receive very low shares (AM, UR and UD). The average weighting structure is the same in both periods. In all the cases, the minimum and maximum values corresponded with the limits set in the specification of the linear programs (3.125 and 9.375, respectively). These results highlight the importance of setting weight constraints in DEA. Without those constraints it is likely that the indicators with low weights (AM, UR and UD) would receive zero weights in the linear programs of many municipalities, which is difficult to justify. On the other hand, the indicators with high weight shares would tend to increase their shares unreasonably.

**Table 5:** Weight shares (%) of the 16 indicators

	2011		2001	
	Average	SD	Average	SD
ASC	8.2	2.3	7.5	2.7
QD	3.6	1.5	4.3	2.2
EM	5.3	2.7	5.3	2.4
AM	4.0	2.1	3.4	1.1
OLE	5.5	2.8	7.6	2.6
UD	3.3	1.0	3.3	0.9
O_3_	9.1	1.3	8.9	1.3
PM_10_	6.0	2.8	4.7	2.4
CRI	6.0	2.7	4.6	2.4
UR	3.4	1.3	4.6	2.6
PME	8.1	2.4	8.5	2.1
CS	7.8	2.5	7.5	2.2
VA	4.5	2.4	6.1	2.7
CSC	6.9	2.4	7.0	2.5
CMS	9.2	1.0	8.6	1.5
CT	9.1	1.2	7.9	2.1

**Fig. 1:**
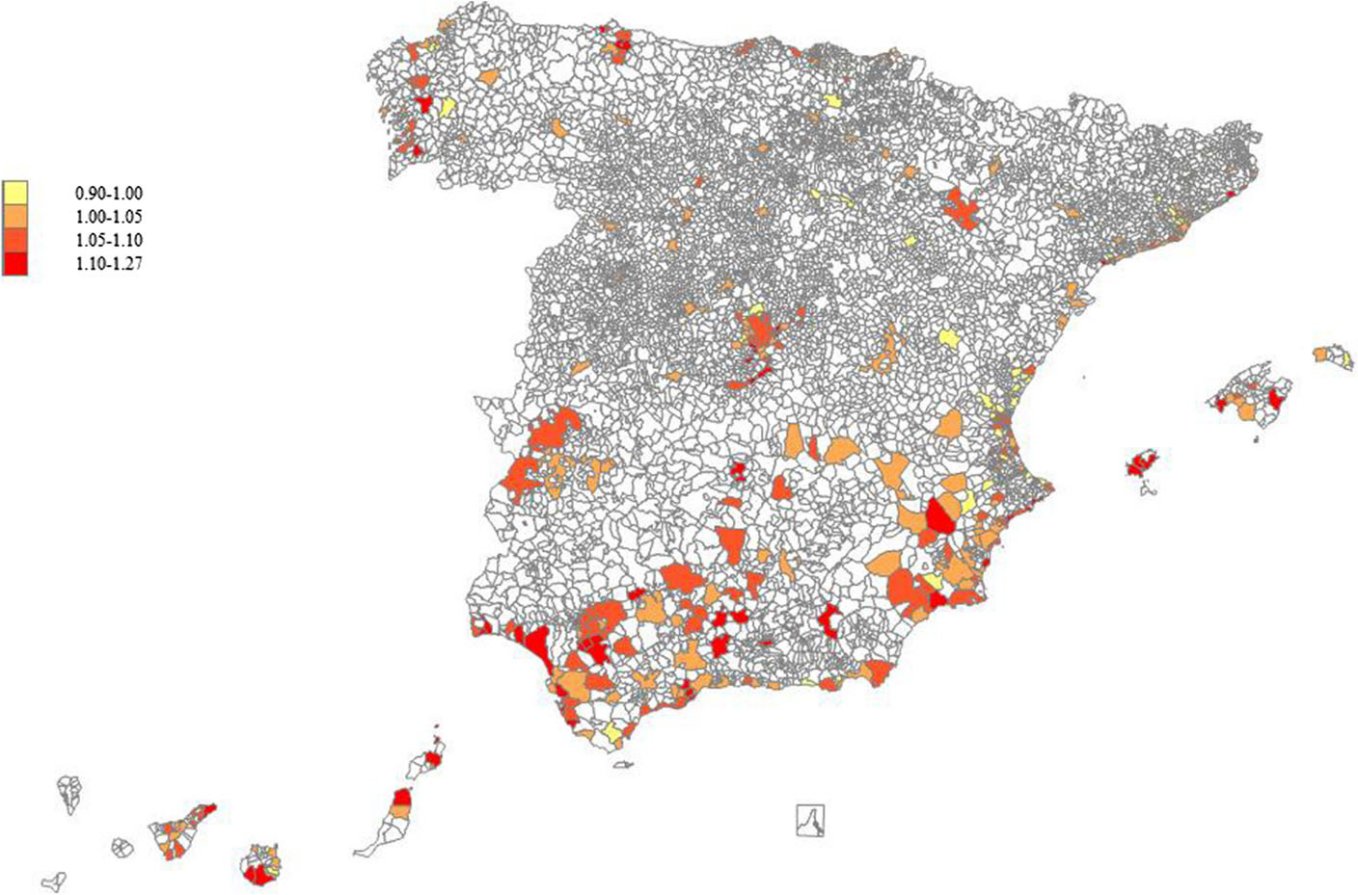
Malmquist index of social progress in Spanish municipalities

Figure 1 shows the geographical distribution of the Malmquist index of social progress in Spain. The yellow tones indicate social regress, while the orange and red tones indicate positive social progress. The map illustrates how the southern regions (including the islands) concentrate the largest progress in QoL conditions. As stated above this is a good result in terms of territorial cohesion, since these regions were also the ones showing the lowest QoL scores in 2001. We also observe dark red areas in the west north, including Galicia, Asturias and Cantabria. In contrast, the yellow tones dominate the Mediterranean regions (Comunidad Valenciana and Cataluña) and some areas in the central north. In the central part of the map, we can see the municipalities in the AC of Madrid. We appreciate how the Southern municipalities, which scored poorly in 2001 have notably improved in 2011, while the opposite occurs in the Northern areas.

Table 6 shows the top 10/bottom 10 ranking of the 81 municipalities which are either provincial capitals, AC capitals or have population over 100,000 with respect to catching-up. Santa Cruz de Tenerife (Canarias) reports the largest improvement in QoL with respect to the frontier with an 8.7% improvement, closely followed by Granada (Andalucía) and Parla (Madrid). In contrast, the other Canarian provincial capital (Las Palmas de Gran Canaria) is in the bottom 10, contributing to the convergence between the big cities of Canarias. Soria (Castilla y León) falls abruptly from the QoL frontier with the most negative trend observed in this subsample. The bottom 10 is dominated by municipalities from Cataluña, while the top ten is dominated by southern municipalities mainly from Andalucía and Madrid. It is surprising the appearance of Vitoria (País Vasco) in the bottom 10 list. With a QoL index of 0.89 in 2001, Vitoria was the municipality with the highest QoL among those over 200,000 population. Even after dropping to 0.86, Vitoria continues as the municipality over 200,000 with the largest QoL index. This case reflects a basic fact in the dynamic evolution of the frontiers. If positive catching-up is observed some of the municipalities with a low QoL index must be improving greatly, while some of the municipalities that were close to the frontier must be stagnating or even reducing their own QoL levels.

**Table 6:** Best and worst large municipalities in terms of catching-up

	AC	QoL index 2001	QoL index 2011	Malmquist index	Catching-up effect	Frontier Shift
Santa Cruz de Tenerife	Canarias	0.75	0.81	1.109	1.087	1.020
Granada	Andalucía	0.78	0.84	1.109	1.084	1.023
Parla	Madrid	0.65	0.71	1.135	1.083	1.047
Ciudad Real	C. Mancha	0.82	0.88	1.111	1.079	1.029
Torrejón de Ardoz	Madrid	0.71	0.76	1.125	1.074	1.047
Cáceres	Extremadura	0.86	0.92	1.091	1.072	1.017
Sevilla	Andalucía	0.71	0.76	1.093	1.072	1.019
Marbella	Andalucía	0.74	0.79	1.090	1.068	1.020
Toledo	C. Mancha	0.87	0.92	1.090	1.068	1.021
Alcobendas	Madrid	0.83	0.89	1.099	1.065	1.032
Palmas de Gran Canaria	Canarias	0.80	0.78	1.011	0.979	1.033
Lleida	Cataluña	0.86	0.84	1.009	0.975	1.035
Vitoria-Gasteiz	País Vasco	0.89	0.86	0.993	0.973	1.020
Algeciras	Andalucía	0.71	0.69	1.031	0.973	1.060
Sabadell	Cataluña	0.81	0.79	1.002	0.969	1.034
Mataró	Cataluña	0.77	0.75	0.996	0.968	1.029
Telde	Canarias	0.73	0.70	1.013	0.968	1.047
Reus	Cataluña	0.82	0.79	0.992	0.960	1.033
Terrasa	Cataluña	0.80	0.76	0.988	0.953	1.036
Soria	C. León	1.00	0.93	0.926	0.931	0.994

If we focus on the ten biggest municipalities, Sevilla and Madrid lead the catching-up movement, with scores of 1.072 and 1.059, respectively. They are followed by Zaragoza (1.054) and Valencia (1.040). In contrast, Las Palmas de Gran Canaria (0.979), Murcia (0.993), Bilbao (1.005), Palma de Mallorca (1.007), Málaga (1.014) and Barcelona (1.017) obtain the lowest scores. Taking into account the complete sample, the municipality with the largest catching-up (and also the largest Malmquist index of social progress) is Mogán, a small Canarian municipality that doubled population from 12,444 to 22,847 within this decade, with an impressive improvement of 1.272. The QoL of this municipality is still well below the QoL frontier (0.76) but made huge progress especially in terms of health and crime records.

In sum, we observe a very positive evolution of the variables measuring the QoL in the Spanish municipalities. This trend is more pronounced in the municipalities that started off in the worst positions in 2001, which has the effect of contributing to positive catching-up. The frontier shift and the catching-up effect almost split into equal parts the social progress estimated with the Malmquist index. Municipalities in southern regions have benefited more with larger improvements, while the Mediterranean ACs have followed the worst dynamic evolution.^24^

## Concluding Remarks

The measurement of QoL in municipalities requires combining information on many different dimensions of life. In this paper, we made a proposal based on eight different domains, which are rooted on previous literature on QoL composite indicators. In the case of Spain, partial indicators for all such dimensions are not readily available at the municipal level. However, we have shown that using microdata from different sources it is possible to construct partial indicators capable of approximating each of the eight dimensions. Our model uses 16 partial indicators, two per dimension. Some are innovative and require complex elaboration from the microdata. For instance, the health indicator of avoidable mortality requires using the complete microdata of mortality with cause of death and to determine which of those causes can be considered avoidable or which of them cannot, on the basis of clinical consensus. Other indicators are easier to obtain, since they are published in the required format (e.g., unemployment).

But if measuring QoL in municipalities at a given point in time is a demanding task, the assessment of its temporal evolution is even more complex. In the first place, comparable indicators must be available for different time periods. In our case, an important number of the indicators comes from the census, which is elaborated every 10 years. This fact restricted the comparison to the census years 2001 and 2011. Unfortunately, the methodology used by the census was slightly modified during this period, which affected some of the indicators used for 2001 in our previous research (González et al. 2011). On the other hand, some new information was included in the 2011 census that was not collected in 2001 (e.g., volunteering activities). For this reason, in this paper a complete new set of indicators was designed and collected that were available in 2001 and 2011 for the 393 municipalities over 20,000 population (this covers 68% of the Spanish population). The second challenge is methodological. Consistent with our non-parametric DEA approach to the measurement of QoL, in this paper we have employed the Malmquist productivity index to gain insight in the evaluation of the dynamic evolution of social progress. In the context of QoL assessment, the Malmquist index can be understood as an index of social progress, which reflects the overall improvement in the partial indicators used to construct the composite indicator of QoL. The Malmquist index of social progress shows an average improvement of 5.4% during the decade. Social progress is observed (on average) in all the ACs in which the Spanish territory is administratively divided. Progress is especially large in Andalucía, Madrid, Aragón, Asturias and Cantabria. The map reveals that southern municipalities (including the islands) dominate the positive trend in the Malmquist index of social progress, while the Mediterranean regions show a worrying trend towards social regress.

The decomposition of the Malmquist index results in an index of catching-up and an index of frontier shift. The catching-up effect measures the extent to which the municipalities are now closer to of farther from the best QoL frontier. The results show, that catching-up has occurred in almost all the ACs and especially in those municipalities that were farther away from the frontier in 2001. This result suggests a trend towards convergence and lower inequality in 2011 regarding QoL variables. Again, Asturias, Madrid, Cantabria and Andalucía lead the positive trend in catching-up. The frontier shift effect measures the movement of the QoL frontier outwards or inwards, due to shared social progress or regress. Our results evidence the global trend to social progress, since the measured frontier shift is around 2.8% and this average is very similar for all the ACs.

A major limitation of our analysis is the restriction in sample size, which only includes the largest municipalities. The exclusion of small rural areas may bias our results when social progress drivers have asymmetric effects on different areas. For instance, it may well be the case that the effects of the housing bubble in the Mediterranean regions had been larger on urban than rural areas. In contrast, the improvements in education may have been larger in urban areas. Moreover, some rural regions (Navarra, Castilla La Mancha, Extremadura) are not well represented in the database even in terms of population. Future research could address these differences between rural and urban areas. A second important limitation of our research is the lack of time variation for two of the variables considered (VA and CS). We think the results obtained may be conservative, at least with respect to the CS variable, since the economic crisis might have deteriorated municipal finances in ways which cannot be captured by our model.

In sum, we have shown how the use and decomposition of the Malmquist index can add new valuable perspectives in the assessment of QoL in Spanish municipalities. Unfortunately, the information available at the municipal level is still scant and depends to a great extent on the census, which is elaborated every 10 years. Thus, the next evaluation of social progress, as done in this paper, for the Spanish municipalities will not be possible until the data of the next census (in 2021) is available.

## Footnotes

1 See Reig-Martinez (2013) and Somarriba et al. (2015) for recent examples.

2 Data obtained online from the official statistics of the Instituto Nacional de Estadística. The poverty line is defined as the 60% of the median of per capita income.

3 Data obtained online from the official statistics of Eurostat. Severe material deprivation is defined as lacking at least four out of a list of 9 concepts which include 1 week of vacation per year, one meal with meat, chicken and fish every two days, having the house at appropriate temperature, capacity to face unexpected expenses, problem in payments related to housing (energy, rent, etc.), an automobile, a phone, a tv, a wash machine

4 Unfortunately, only the initial impact of the crisis can be evaluated in this paper, since the worse effects of it came after 2011. The first years of the crisis (2009–2011) were less severe on the population. Family support networks and unemployment insurance acted as softeners of the effects of the crisis. The crisis and its effects worsen after 2011. Therefore, the analysis of the period 2011–2015 could show a very different picture than the one reported in this paper. However, since many of the municipal data that we need come from the census, we will not be able to repeat this analysis until 2021.

5 Other authors have focused on the study of QoL in the Spanish regions or provinces, levels of analysis in which statistical information is better developed (Murias et al. 2006; Jurado and Perez-Mayo 2012).

6 The ESS sponsorship group proposes a 9^th^ dimension (Overall Experience of Life) to account for the subjective perception of the own QoL. So does the Better Life Index of the OECD. While we sympathize with the life satisfaction dimension, in this study we only have access to objective dimensions (not self-reported perceptions). Therefore, our QoL index will be constructed in the tradition of the Scandinavian welfare research (Erikson 1974, 1993) that bases QoL exclusively on objective indicators. It can also be understood in terms of the capabilities approach (Sen 1993).

7 We briefly review these indicators here. More detailed explanations can be found in Gonzalet et al. (2016).

8 This index ranges from 0 to 100 and takes into account factors of the buildings as the age of construction, tumbledown status, hygienic conditions, running water, indoor toilet, accessibility, heating, etc.

9 Mortality microdata include the complete registry of deaths including the cause of death, age and residence. The microdata identifying the municipality of residence are not publicly available. We thank the INE for providing these data for our research.

10 Death rates have been found to correlate highly with life satisfaction at the county level (Lawless and Lucas 2011).

11 To be exact, we used the data of population under 75, since most cases of death are only considered to be avoidable for individuals below that cut-off age.

12 The WHO also stresses the importance of Nitrogen Dioxide (NO_2_) and Sulfur Dioxide (SO_2_). Unfortunately, data for these two elements were not available for the entire sample.

13 The data for País Vasco are publicly available online. In the case of Cataluña the data were provided by the Autonomous Government upon request for this research.

14 This variable was obtained from the municipal database of CajaEspaña.

15 To compute this index, La Caixa takes into account the population, number of phones, automobiles, trucks and vans, banking offices and retail activities. In order to make this index comparable across municipalities we divided it by the population.

16 The raw data distinguishes between two destinations (job or school). Our variable is the arithmetic average of both. We also must indicate that INE does not compute an index associated with these variables. Instead the report includes the percentage of people on seven intervals that go from "less than 10 min" to "more than 90 min". We took mark classes in the mean of the intervals (90 for the last interval) and weighted each class mark by the percentage of population within the interval. The weighted sum can be interpreted as the average time employed to get to the school or job and is the variable used in this paper.

17 Many of these variables (or similar indicators) are proposed by the EU Sponsorship Group on measuring social progress and by the OECD Better Life Index. For instance, the unemployment rate, excess mortality, quality of dwellings, overall level of education, air quality (PM_10_, O_3_), voluntary work, crime rates and polls numbers can be found in very similar or identical form. There are also indicators which are similar to the average socioeconomic condition and commercial market share. Even though our selection of indicators is constrained by data availability at the municipal level, we believe it offers a good description of the QoL dimensions, as they are specified in those initiatives.

18 To avoid being repetitive, in this paper we will report the results obtained only with the first approach. Both qualitatively and quantitatively, these results are very similar with the ones obtained with the second approach.

19 DEA was developed to measure efficiency in production, where a set of inputs (resources) are combined to produce a set of outputs (products and services).

20 The CS indicator is a special case, since it is the only variable in which negative values are possible (the cash surplus is indeed negative in many municipalities). In the case of this variable, we normalized the values to positive before the transformation, by adding the minimum in absolute value plus a small amount, in order to avoid 0 values in the transformed variable.

21 It is interesting to notice that since the input variable is a vector of 1, there is no difference between the alternative DEA specifications with either constant or variable returns to scale. This is an additional advantage of the Cherchye et al. (2004) transformation.

22 Since these weight limits are arbitrary, we checked the robustness of the results by modifying the weight constraints between different limits. We followed González et al. (2016; 2017) by computing the scores with 30%, 40%, 50%, 60% and 70% common weight and also equal weighting. For all the indexes computed, the Pearson and Spearman correlation between each pair of different weighting limits is above 0.90. Therefore, our results do not change quantitatively or qualitatively when different percentages of common weight are selected. Correlations are also high with respect to equal weighting (above 0.8 in all cases).

23 The political-administrative structure of Spain clusters municipalities into provinces and provinces into Autonomous Communities (ACs). There are 17 ACs and two autonomous cities (Ceuta and Melilla). The averages contained in the table are weighted by the population of the municipality.

24 Given that two of the 16 partial indicators of QoL don’t have time variation (are only observed in 2011), we repeated the complete analysis using only the other 14 indicators to obtain the Malmquist index of social progress and the decomposition. Even though there are some variations in the exact numbers, the results are qualitatively very similar. The geographic distribution of QoL and catching-up effects is identical.
